# Long-Term Blood Pressure Control Effect of Celiac Plexus Block with Botulinum Toxin

**DOI:** 10.3390/toxins8020051

**Published:** 2016-02-19

**Authors:** Sung Hyun Lee, Dae Hwan Lim, Ju Ho Lee, Kiyuk Chang, Jung Min Koo, Hue Jung Park

**Affiliations:** 1 Department of Anaesthesiology & Pain Medicine, Seoul St. Mary’s Hospital, College of Medicine, The Catholic University of Korea, Seoul 06591, Korea; 4321hoho@catholic.ac.kr (S.H.L.); ldh7415369@naver.com (D.H.L.); sonofkp@naver.com (J.H.L.); miniyaa623@gmail.com (J.M.K.); 2 Division of Cardiology, Department of Internal Medicine, Seoul St. Mary’s Hospital, College of Medicine, The Catholic University of Korea, Seoul 06591, Korea; Kiyuk@catholic.ac.kr

**Keywords:** celiac plexus block, resistant hypertension, botulinum toxin

## Abstract

Celiac plexus block (CPB) is one of the main treatment options for patients resistant to conventional antihypertensive drugs. We present a case of resistant hypertension (RHTN) that was treated with CPB using botulinum toxin. An 18-year-old male patient with RHTN, who suffered from persistent hypertension even after combination therapy and a renal denervation procedure, was referred to our pain center for CPB. CPB using botulinum toxin following the use of only local anesthetics resulted in control of systolic blood pressure (BP) at ~150 mmHg for at least three months.

## 1. Introduction

Resistant hypertension (RHTN) is defined as uncontrolled blood pressure despite the use of three or more antihypertensive agents, including diuretics. Patients with RHTN have an increased risk of cardiovascular complications, such as myocardial infarction, congestive heart failure, arrhythmia, stroke, and chronic kidney disease, and an increased mortality rate compared with those with controlled hypertension [1]. The sympathetic nervous system (SNS) is critical for achieving blood pressure homeostasis. Specifically, sympathetic nervous system activity innervating the splanchnic circulation is increased in early stage hypertension. The AngII-salt models of hypertension have revealed that sympathetic innervation of the splanchnic circulation may be especially important in the development of hypertension [2].

The celiac plexus contains the preganglionic sympathetic efferent nerve fibers from splanchnic nerves (greater splanchnic, lesser splanchnic and least splanchnic nerves). The postganglionic nerves from the celiac plexus accompany the splanchnic blood vessels to their respective visceral structures [3,4]. On account of the anatomic and functional connection, the celiac plexus has an influence on the splanchnic blood vessels. Studies on celiac ganglionectomy in rats have shown a decrease in splanchnic norepinephrine (NE) and an attenuated hypertensive response to angiotensin II [5].

Based on these results, a celiac plexus block can be considered an interventional therapy for resistant hypertension.

## 2. Case Description

**Figure 1:**
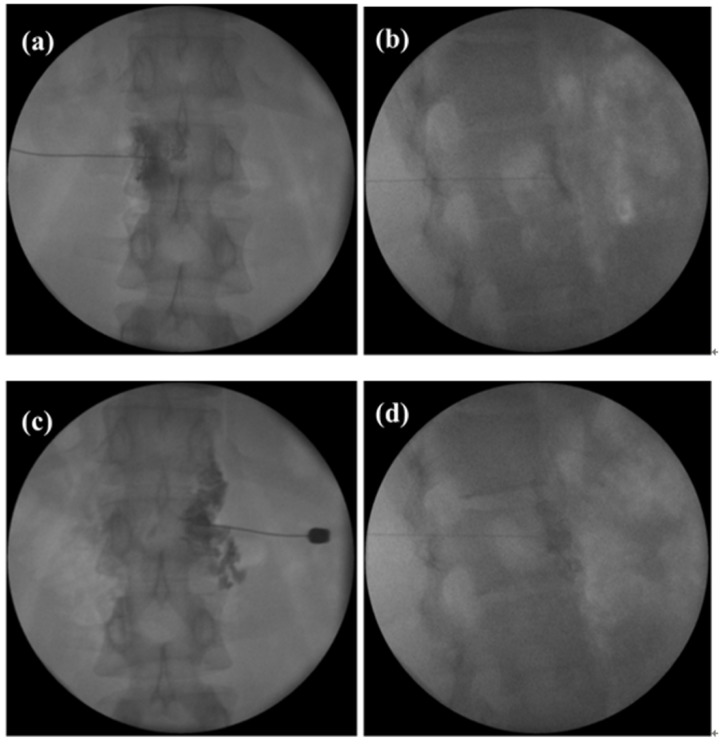
**Left:** celiac plexus block with botulinum toxin Anteroposterior (AP) view (**a**); Lateral view (**b**); **Right:** celiac plexus block with botulinum toxin AP view (**c**); Lateral view (**d**).

An 18-year-old male patient, who was diagnosed with uncontrolled hypertension four years ago, was referred to our pain center by a cardiologist. Four antihypertensive drugs were used (nifedipine 30 mg, chlorthalidone 25 mg, doxazosin 4 mg, and minoxidil 5 mg). In addition, irbesartan 150 mg, spironolactone 25 mg, verapamil 240 mg, and carvedilol 25 mg were administered. However, his hypertension could not be controlled; his systolic BP was >170–180 mmHg, occasionally >200 mmHg. The cardiologist at our institution evaluated the causes of hypertension to exclude secondary hypertension, and concluded that he had essential hypertension. The cardiologist considered that the main factor in the resistance to controlling hypertension was obesity (weight: 106 kg, height: 177 cm, body mass index (BMI): 33.8). However, the weight loss strategy failed. Two years ago, when the patient was 16 years old, the cardiologist tried a new approach, the renal denervation procedure. However, there was no significant improvement in his blood pressure. The patient was referred to our pain center for a celiac plexus block (CPB) six months ago and we performed a prognostic CPB with local anesthetics (1% lidocaine 10 cc per side). We performed a fluoroscopic-guided CPB using the bilateral paravertebral posterior approach. The needle was advanced alongside the vertebral body to avoid the transverse process, visceral organs, and vascular structures. After diffusion of contrast material was confirmed, local anesthetics were injected. One h after the procedure, the systolic and diastolic BP dropped to ~150 mmHg and ~90 mmHg, respectively. The BP was maintained at a similar level for four days. It was checked hourly for six hours and four times a day for the next four days. Then the systolic BP increased to 170 mmHg. This result confirmed the short-term effect of the CPB procedure in terms of a decrease in BP. We planned CPB with botulinum toxin at the next follow-up and performed CPB with Botox^®^ (botulinum toxin 50 IU, two vials containing a total of 100 IU, Allergan Inc., Irvine, CA, USA) in the patient (Figure 1). The patient’s systolic BP decreased to ~150 mmHg within 30 min.

During the one-month observation period after CPB using Botox, the patient’s systolic and diastolic BP were controlled at ~150/90 mmHg using the medications mentioned above, with the exception of on one occasion (BP was 170/100 mmHg). Three months after the first injection, we performed a second CPB using the same dose of botulinum toxin (Table 1). The patient’s systolic BP declined to ~150 mmHg and has been controlled at an appropriate level for four months.

## 3. Discussion

This case shows that resistant hypertension can be controlled by a celiac plexus block (CPB) using botulinum toxin for three to four months. The results of many studies had shown that CPB can be performed safely by experienced clinicians. Transient hypotension, diarrhea and procedural pain for several days can happen after CPB. Those are minor complications which could be tolerated with conservative care. Serious complications are uncommon when the procedure is done by a skilled clinician [3,6].

Studies on the pathophysiology of hypertension have shown an abnormal increase in vascular tone induced by the sympathetic nervous system (SNS). Moreover, a change in the renal system induced by the renal SNS facilitates sodium reabsorption, renin secretion and renal vasoconstriction [7]. The interventional approach in this patient, renal denervation and CPB, was based on attenuating SNS activity. CPB was more effective than renal denervation in this patient. In rats, surgical celiac ganglionectomy resulted in a greater decrease in tissue norepinephrine (NE) content compared to renal denervation (RDx). Celiac ganglionectomy resulted in a significant reduction in the NE contents of the left and right kidneys, spleen, small intestine, and liver. RDx resulted in a significant reduction in the NE contents of the left and right kidneys, but did not affect that of the spleen, small intestine, or liver. The decrease in total NE was greater following celiac ganglionectomyn [5,8,9]. Generally, a local anesthetic agent is used in CPB, and alcohol (50% or 100%) and phenol (6% or 12%) are used for celiac plexus neurolysis. In some case reports, alcohol induced neuritis, which spread into the lumbosacral plexus and sacral canal [10]. We did not use alcohol due to this side effect [11]. In several cases, pulsed radiofrequency ablation was used for neuromodulation. However, radiofrequency ablation was not considered to be appropriate for the long-term effect of CPB on account of the size of the plexus. The celiac plexus is the largest visceral plexus, ranging from 0.5 to 4.5 cm in diameter [4]. The radiofrequency-induced lesion is not large enough to cover the celiac plexus. An effect can be expected if the electrode is placed in a suitable position and not distally [12,13]. In this patient, Botox^®^ was used. Botox^®^ is a widely used therapeutic protein that inhibits release of the neurotransmitter acetylcholine from the presynaptic membrane. This results in the complete blockage of signals to neuromuscular junctions in a broad region, such as the celiac plexus. The effect of Botox^®^ in sympathectomy, excluding thoracic sympathectomy, for hyperhidrosis was of a six-and-a-half-month duration [14]. However, there are no data on the duration of the effect of Botox^®^ in CPB; hence, further prospective studies are required to identify the duration and efficacy of Botox^®^.

In conclusion, this case suggested CPB to be a treatment option for resistant hypertension. However, this supposition should be confirmed in further work. Determination of the mechanism or the electrophysiology of neurotransmitter dysregulation through the celiac plexus leading to resistant hypertension will facilitate the application of CPB for the treatment of resistant hypertension.

## Figures and Tables

**Table 1:** The process of antihypertensive therapy.

Time Progress	4 Years Ago	2 Years Ago	7 Months Ago	4 Months Ago
Experimental therapy conducted.	Prescription of antihypertensive medication. Recommending the patient to lose his weight.	Catheter-based renal denervation procedure by cardiologist.	The first trial of celiac plexus block with Botox^®^ after confirming the short time effect of celiac plexus block with 1% lidocaine.	A Second celiac plexus block with Botox^®^.

## References

[1] Daugherty S.L., Powers J.D., Magid D.J., Tavel H.M., Masoudi F.A., Margolis K.L., O’Connor P.J., Selby J.V., Ho P.M. (2012). Incidence and prognosis of resistant hypertension in hypertensive patients. Circulation.

[2] Osborn J.W., Fink G.D., Kuroki M.T. (2011). Neural mechanisms of angiotensin II–salt hypertension: Implications for therapies targeting neural control of the splanchnic circulation. Curr. Hypertens. Rep..

[3] Erdine S. (2005). Celiac ganglion block. Agri.

[4] Kambadakone A., Thabet A., Gervais D.A., Mueller P.R., Arellano R.S. (2011). CT-guided celiac plexus neurolysis: A review of anatomy, indications, technique, and tips for successful treatment. Radiographics.

[5] Li M., Galligan J., Wang D., Fink G. (2010). The effects of celiac ganglionectomy on sympathetic innervation to the splanchnic organs in the rat. Auton. Neurosci. Basic Clin..

[6] O’Toole T.M., Schmulewitz N. (2009). Complication rates of EUS-guided celiac plexus blockade and neurolysis: Results of a large case series. Endoscopy.

[7] Prince E.A., Murphy T.P., Hampson C.O. (2012). Catheter-based arterial sympathectomy: Hypertension and beyond. J. Vasc. Interv. Radiol..

[8] Lohmeier T.E., Hildebrandt D.A., Dwyer T.M., Barrett A.M., Irwin E.D., Rossing M.A., Kieval R.S. (2007). Renal denervation does not abolish sustained baroreflex-mediated reductions in arterial pressure. Hypertension.

[9] King A.J., Osborn J.W., Fink G.D. (2007). Splanchnic circulation is a critical neural target in angiotensin II salt hypertension in rat. Hypertension.

[10] Vranken J.H., Zuurmond W.W., Van Kemenade F.J., Dzoljic M. (2002). Neurohistopathologic findings after a neurolytic celiac plexus block with alcohol in patients with pancreatic cancer pain. Acta Anaesthesiol. Scand..

[11] Lim S.J., Park H.J., Lee S.H., Moon D.E. (2010). Ganglion impar block with botulinum toxin type A for chronic perineal pain-A case report. Korean J. Pain.

[12] Cosman E.R., Dolensky J.R., Hoffman R.A. (2014). Factors that affect radiofrequency heat lesion size. Pain Med..

[13] Bogduk N. (2006). Pulsed radiofrequency. Pain Med..

[14] Vorkamp T., Foo F.J., Khan S., Schmitto J.D., Wilson P. (2010). Hyperhidrosis: Evolving concepts and a comprehensive review. Surgeon.

